# Molecular subtypes and a prognostic model for hepatocellular carcinoma based on immune- and immunogenic cell death-related lncRNAs

**DOI:** 10.3389/fimmu.2022.1043827

**Published:** 2022-11-21

**Authors:** Mingang He, Wenchao Gu, Yang Gao, Ying Liu, Jie Liu, Zengjun Li

**Affiliations:** ^1^ Department of Gastrointestinal Surgery, Shandong Cancer Hospital and Institute, Shandong First Medical University and Shandong Academy of Medical Sciences, Jinan, China; ^2^ Department of Pulmonary and Critical Care Medicine, Shanghai Pudong New Area People’s Hospital, Shanghai, China; ^3^ Department of Pathology, Shandong Cancer Hospital and Institute, Shandong First Medical University and Shandong Academy of Medical Sciences, Jinan, China; ^4^ Cancer Center, Shandong Public Health Clinical Center, Public Health Clinical Center Affiliated to Shandong University, Jinan, China

**Keywords:** hepatocellular carcinoma, lncRNAs, immunogenic cell death, biomarkers, clinical prognosis

## Abstract

**Background:**

Accumulating evidence shows that immunogenic cell death (ICD) enhances immunotherapy effectiveness. In this study, we aimed to develop a prognostic model combining ICD, immunity, and long non-coding RNA biomarkers for predicting hepatocellular carcinoma (HCC) outcomes.

**Methods:**

Immune- and immunogenic cell death-related lncRNAs (IICDLs) were identified from The Cancer Genome Atlas and Ensembl databases. IICDLs were extracted based on the results of differential expression and univariate Cox analyses and used to generate molecular subtypes using ConsensusClusterPlus. We created a prognostic signature based on IICDLs and a nomogram based on risk scores. Clinical characteristics, immune landscapes, immune checkpoint blocking (ICB) responses, stemness, and chemotherapy responses were also analyzed for different molecular subtypes and risk groups.

**Result:**

A total of 81 IICDLs were identified, 20 of which were significantly associated with overall survival (OS) in patients with HCC. Cluster analysis divided patients with HCC into two distinct molecular subtypes (C1 and C2), with patients in C1 having a shorter survival time than those in C2. Four IICDLs (TMEM220-AS1, LINC02362, LINC01554, and LINC02499) were selected to develop a prognostic model that was an independent prognostic factor of HCC outcomes. C1 and the high-risk group had worse OS (hazard ratio > 1.5, *p* < 0.01), higher T stage (*p* < 0.05), higher clinical stage (*p* < 0.05), higher pathological grade (*p* < 0.05), low immune cell infiltration (CD4^+^ T cells, B cells, macrophages, neutrophils, and myeloid dendritic cells), low immune checkpoint gene expression, poor response to ICB therapy, and high stemness. Different molecular subtypes and risk groups showed significantly different responses to several chemotherapy drugs, such as doxorubicin (*p* < 0.001), 5-fluorouracil (*p* < 0.001), gemcitabine (*p* < 0.001), and sorafenib (*p* < 0.01).

**Conclusion:**

Our study identified molecular subtypes and a prognostic signature based on IICDLs that could help predict the clinical prognosis and treatment response in patients with HCC.

## Introduction

Liver cancer is the second most common cause of cancer-related deaths worldwide (1), with hepatocellular carcinoma (HCC) being the most common histological type (2). Despite advancements in HCC treatment, the 5-year survival rate of patients with HCC remains less than 30% owing to complex etiology and high degree of heterogeneity (3). A novel prognostic indicator is, thus, required to accurately predict prognosis and guide appropriate treatment for patients with HCC.

Long non-coding RNAs (lncRNA) are RNA transcripts that are longer than 200 nucleotides; however, they do not encode proteins (4). LncRNAs are appealing biomarkers for cancer diagnosis and prognosis because of various reasons. First, lncRNA expression varies greatly across disease stages, diseases, and tissues; thus, it can better represent disease features (5). Second, lncRNA can regulate gene transcription, post-transcriptional modification, and epigenetic expression (6, 7), which correlates more closely with tumor progression.

Numerous studies have examined the clinical relevance of lncRNA within tumors, including HCC (8). LncRNA has been recently revealed to regulate the expression of genes encoding anticancer immunity proteins (9). Tumor immune cell infiltration also depends on lncRNA (10). For example, NRON sequesters phosphorylated NFAT in the cytoplasm and maintains T cell resting states (11). Antisense lncRNA SATB2-AS1 regulates the expression of SATB2, inducing the expression of TH1 chemokines CXCL9 and CXCL10 and initiating the transport of effector T cells (12). Moreover, GAS5 plays a crucial role in the growth arrest of T cells and non-transformed lymphocytes (13), and FENDRR promotes inflammatory and antitumor immunity by controlling tumor cell immunogenicity and proliferation (14). In patients with cancer, LIMIT correlates with MHC-I, tumor-infiltrating T cells, and checkpoint blockade response (15).

Immunotherapy has received increasing research attention in recent years. Immune checkpoint inhibitors yielded promising results in several clinical trials for HCC (16–18). They work primarily to prevent the activation or effects of T cells (19). An immune checkpoint inhibitor, nivolumab (anti-PD-1), was the first FDA-approved treatment for HCC. It reduces the number of unresponsive T cells while increasing CD38-expressing activated T cell counts (20). In 20% of HCC patients, pembrolizumab has shown efficacy in rejuvenating exhausted T cells and restoring their antitumor functions by inhibiting the PD-1 pathway (21, 22). Similarly, ipilimumab targets CTLA4 receptors and blocks their interaction with CD80/86, thereby allowing T cells to release suppressive signaling and become cytotoxic (23). Increasing evidence shows that cancer antigens released by immunogenic cell death (ICD) can boost cytotoxic T cell responses, potentially improving immunotherapy (24–28).

ICD is a form of cell death that elicits an immune response against the antigens of dead or dying cells, mostly cancerous cells (29, 30). ICD may activate danger-signaling pathways mediated by surface calreticulin/heat-shock proteins, secreted ATP, or HMGB1 (31–33). Extensive preclinical studies have identified ICD as a significant predictor of solid antitumor immunity (34, 35). ICD for biomarker discovery offers considerable advantages because it allows simultaneous integration of several immune-related pathways, such as those of danger signaling and effector T cell infiltration/activity (36, 37). The induction of ICD may become a more effective approach in cancer immunotherapy since it allows the eradication of tumors that had previously escaped the immune system (38).

As a single or combined agents with chemotherapy that induces ICD, TLR7 agonists have been used as anticancer immunotherapeutic agents (39, 40). Chemotherapy-induced ICD can convert malignant cells into vaccines and increase T cell priming, thereby facilitating T cell-mediated destruction of residual cancer cells (41, 42). Moreover, conventional NIR-PIT induces ICD that drives naive T cells to differentiate into effectors by maturing dendritic cells (43, 44). Thus, ICD plays a crucial role in triggering anticancer immune responses, particularly T cell response. Further research into the interactions between ICD and immunity may provide new insights into tumor immunotherapy.

In this study, molecular subtypes and a lncRNA prognostic risk model combining ICD and immunity were constructed to predict the immune microenvironment, prognosis, and response to immunotherapy and chemotherapy in HCC. Our findings pave the development of treatment strategies that will benefit HCC patients.

## Materials and methods

### Data acquisition, differential expression analysis, and intersection identification

The Cancer Genome Atlas (TCGA) (https://portal.gdc.cancer.gov/) database was used to download RNA sequencing (RNA-seq) data and clinical information. Additionally, we downloaded gene transfer format files from Ensembl (http://asia.ensembl.org) to identify the lncRNAs from mRNAs. The limma package (v3.40.2) (45) was used to identify differentially expressed lncRNAs between normal and tumor tissues with *p* < 0.05 and |log_2_ fold change (FC)| > 1. Next, we downloaded a list of immune-related genes (IRGs) from the ImmPort database (http://www.immport.org ), which were used in co-expression analysis to identify immune-related lncRNAs (irlncRNAs). Immunogenic cell death-related lncRNAs (icdrlncRNAs) were identified using co-expression analysis of 33 recognized ICD-related genes (ICDRGs) extracted from a large-scale meta-analysis (46). Immune-related and immunogenic cell death-related lncRNAs (IICDLs) were generated from the intersection of two DElncRNA datasets. Thereafter, Spearman’s correlation analysis was used to describe gene–gene correlation. Gene intersection analysis was performed using the ggplot2 package (v3.3.3).

### Cluster analysis

A univariate Cox regression analysis was used to screen for prognosis-related IICDLs. A cluster analysis was performed using the package ConsensusClusterPlus (v1.54.0) to identify IICDLs related to molecular subtypes. Heat map clustering was performed using the pheatmap package (v1.0.12). Kaplan–Meier (KM) analysis was used to compare the prognosis of both clusters. Stack graphs were used to visualize the correlation between clusters and clinical parameters, and chi-square tests were used to analyze them. Visualization was performed using the ggplot2 package (v3.3.3).

### Differential expression analysis and enrichment of clusters

The limma package was used to analyze the differential expression of mRNA between clusters 1 and 2. The threshold for differential mRNA expression was defined as *p* < 0.05 and |log_2_ FC| > 0.2. To analyze the biological functions of potential mRNAs, the ClusterProfiler package (v1.54.0) was used to perform Gene Ontology (GO) and Kyoto Encyclopedia of Genes and Genomes (KEGG) analyses. The ggplot2 package (v3.3.3) was used for visualization.

### Building the prognostic signature

We used the prognostic IICDLs to construct a prognostic model using LASSO Cox regression analysis *via* the glmnet package (v4.1-2). Based on the regression coefficient (β) derived from the multivariate Cox regression analysis, the following formula was used to construct a prognostic signature: risk score = (β_lncRNA1_* expression level of lncRNA_1_) + (β_lncRNA2_* expression level of lncRNA_2_) + … + (β_lncRNAn_* expression level of lncRNA_n_). Each patient was assigned a risk score using this formula in TCGA-HCC cohorts. Patients were categorized based on the median risk score into low- and high-risk subgroups, and overall survival (OS) times were compared between the two groups using KM analysis. A time-related receiver operating characteristic (ROC) analysis was performed using the timeROC package (v0.3) to evaluate the prognostic ability of the risk model.

### Construction and evaluation of a predictive nomogram

Both univariate and multivariate Cox regression analyses were performed to test whether the prognostic models are independent of conventional clinical characteristics. A nomogram was developed based on all independent prognostic factors to assess the 1-, 3-, and 5-year survival of patients with HCC (47). Calibration plots were constructed as part of an internal validation process to ensure the predictive accuracy of the nomogram. Nomogram performance was also evaluated using time-dependent ROC analysis. Decision curve analysis (DCA) was performed to determine the clinical net benefit (48).

### Identifying somatic mutations

Data on the somatic mutations in the HCC samples were obtained from TCGA GDC Data Portal in a mutation annotation format. The Maftools package (v2.6.05) was used to visualize and summarize the mutated genes using waterfall plots.

### Identifying the immune landscape

TIMER (https://cistrome.shinyapps.io/timer/) is a web-based resource for estimating the abundance of tumor-infiltrating immune cells (B cells, CD4 and CD8 T cells, neutrophils, macrophages, and dendritic cells) (49). TIMER deduces the abundance of TIICs by deconvolution, Monte Carlo simulations, orthogonal estimates TIMER deduces the abundance of tumor-infiltrating immune cells from gene expression profiles based on a deconvolution method validated by Monte Carlo simulations, orthogonal estimates from DNA methylation-based inferences, and pathological assessments. The abundance of immune cells in each tumor sample was analyzed using TIMER. Eight common immune checkpoint genes (ICGs; *SIGLEC15*, *TIGIT*, *CD274*, *HAVCR2*, *PDCD1*, *CTLA4*, *LAG3*, and *PDCD1LG2*) were compared across molecular subtypes and risk groups. Higher Tumor Immune Dysfunction and Exclusion (TIDE) scores were associated with a shorter survival and poorer immune checkpoint blocking (ICB) treatment response. Using the TIDE database, we calculated the TIDE scores for molecular subtypes and risk groups in TCGA. Microsatellite instability (MSI) may be a predictive biomarker of immunity to immune checkpoint inhibitors (50). Therefore, the MSI score was calculated for each sample in TCGA-HCC cohorts to compare the high- and low-risk patients.

### Analyses of stemness and drug susceptibility

The stemness of cancer cells has recently been recognized as a valuable predictive or prognostic factor (51–53). Thus, we used one-class logistic regression (OCLR) to compare stemness for clusters and risks in TCGA (54). Based on the half-maximal inhibitory concentration (IC_50_) of commonly used chemotherapeutics, including doxorubicin (55), 5-fluorouracil (56), gemcitabine (57), and sorafenib (58), we investigated the molecular subtypes and risk groups associated with them.

### Validation of IICDL expression *in vivo* and *vitro*

The Cancer Cell Line Encyclopedia (CCLE) (https://portals.broadinstitute.org/ccle) database was used to obtain the cell line IICDL expression matrix of tumors. IICDL expression in the HCC tissues and healthy liver tissues was analyzed in TCGA (https://portal.gdc.cancer.gov/), lnCAR (http://lncar.renlab.org/ ), and Gene Expression Profiling Interactive Analysis (GEPIA) (http://gepia.cancer-pku.cn/) databases.

### Statistical analysis

The Student’s t-test was used to compare gene expression between tumor and adjacent non-tumor tissue, and the Mann-Whitney U test was used to evaluate proportional differences. K-M analysis was performed to compare the OS between the risk groups and the subtypes *via* the log-rank test. A multivariate and univariate Cox regression analysis was used for the independent prognosis analysis. Wilcox test was used to compare immune scores between two groups. Statistical analyses were conducted using the R (v4.0.3) software. A significance level of 0.05 was used for all tests. We did not adjust P values for multiple testing (59, 60).

## Results

### Identification of differentially expressed irlncRNAs and icdrlncRNAs and their intersection

**Figure 1:**
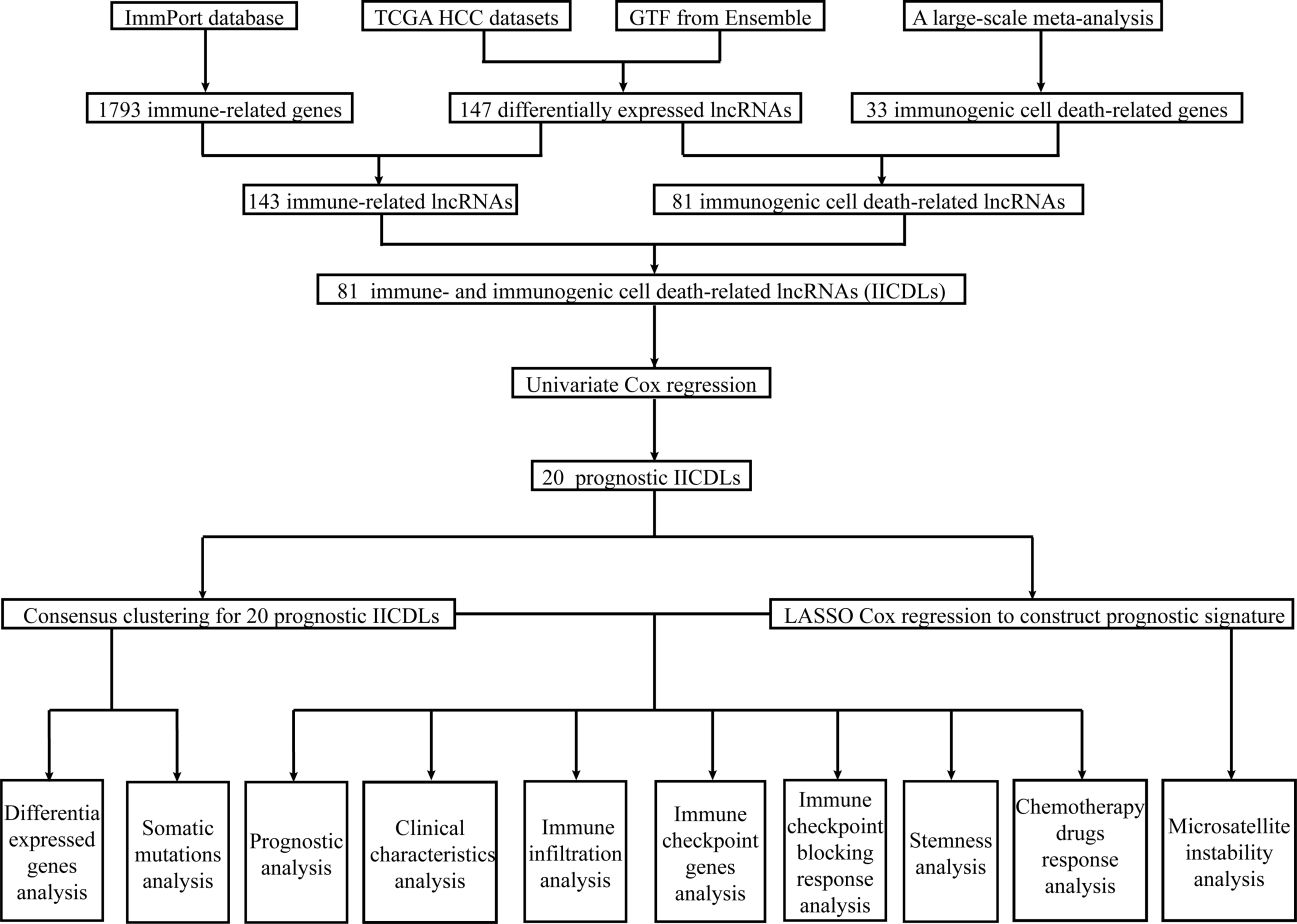
Workflow of the study.

**Figure 2:**
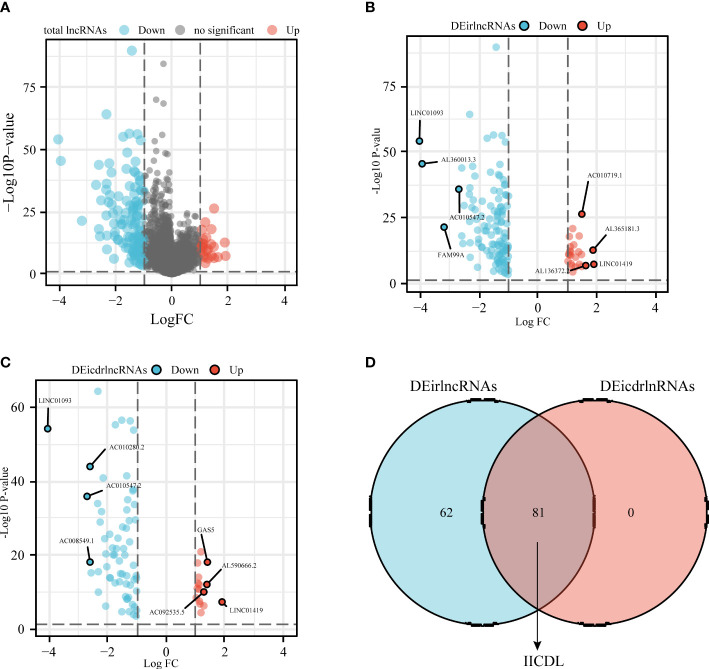
Identification of differentially expressed IICDLs in TCGA **(A)** Identification of differentially expressed lncRNAs in TCGA-HCC cohort. Volcano diagram showing DEirlncRNAs **(B)** and DEicdrlncRNAs **(C)**. **(D)** Intersection of the two sets of differentially expressed lncRNAs. DEirlncRNAs: differentially expressed immune-related lncRNAs; DEicdrlncRNAs, differentially expressed immunogenic cell death-related lncRNAs; IICDLs, immune-related and immunogenic cell death-related lncRNAs; TCGA, The Cancer Genome Atlas.

**Figure 1** shows the flow diagram of this study. TCGA database, which contains 50 standard samples and 371 tumor samples, was used to obtain the HCC RNA-seq data. Based on the Ensembl gene annotation file, 16,013 lncRNA RNA-seq were obtained, and 147 differentially expressed lncRNAs (DElncRNAs) were initially identified, 25 of which were upregulated and 122 downregulated (**Figure 2A**). There were 143 differentially expressed irlncRNAs (DEirlncRNAs), 24 of which were upregulated and 119 downregulated (**Figure 2B**). Moreover, of the 81 differentially expressed icdrlncRNAs (DEicdrlncRNAs), 16 were upregulated and 65 were downregulated (**Figure 2C**). The intersection of these two DElncRNA datasets was defined as IICDLs, comprising 81 lncRNAs (**Figure 2D**).

### Identification of clusters associated with IICDLs

**Figure 3:**
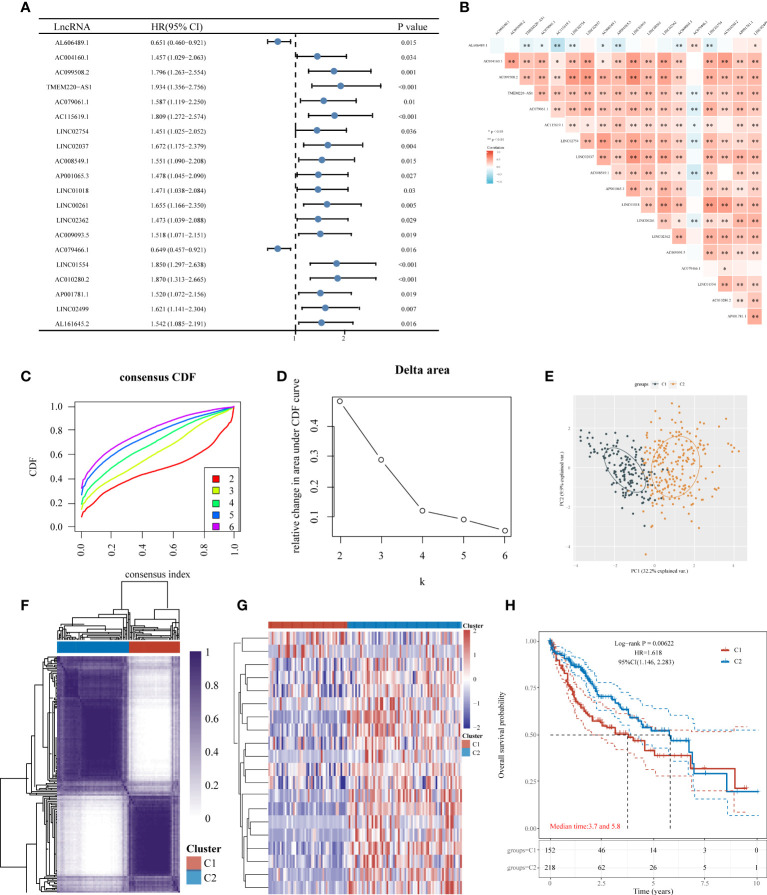
Consensus clustering of HCC molecular subgroups based on IICDLs. **(A)** Forest plot of 20 prognostic IICDLs using univariate Cox analysis. **(B)** Correlations between the 20 genes. CDF curve **(C)**, delta area curve **(D)**, PCA plot **(E)**, and heat map **(F)** of consensus clustering. **(G)** Heat map of IICDL expression in different subtypes. **(H)** KM survival curve of various subgroups in TCGA data sets. CDF, cumulative distribution function; PCA, principal component analysis; KM, Kaplan–Meier. (*P < 0.05; **P < 0.01).

**Figure 4:**
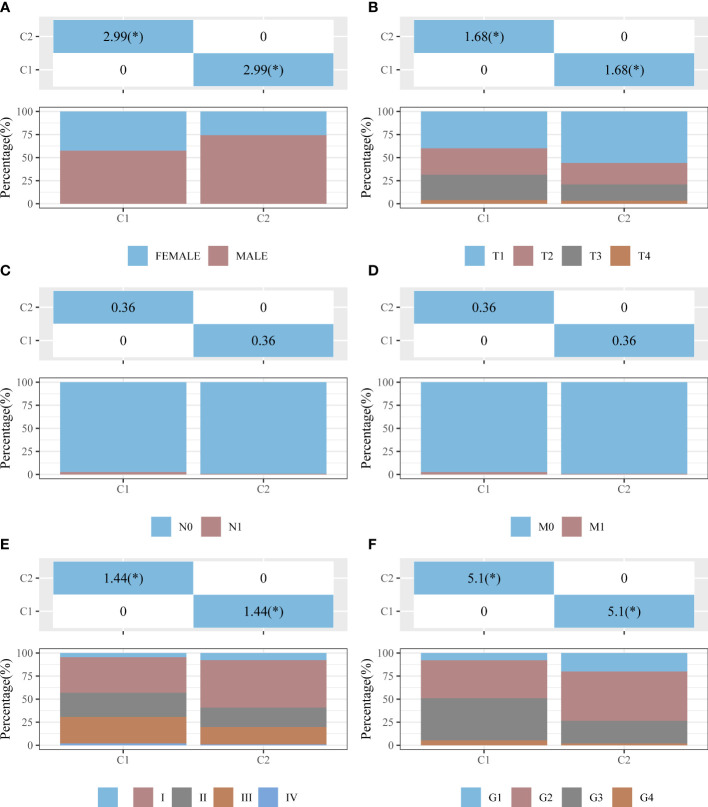
Distribution of clinical characteristics between clusters C1 and C2. Distributions in terms of sex **(A)**, T stage **(B)**, N stage **(C)**, M stage **(D)**, clinical stage **(E)**, and pathological grade **(F)** of clusters C1 and C2. The horizontal axis represents a group of samples, whereas the vertical axis represents the percentage of clinical information contained in the corresponding grouped samples. In the table, the *p*-value (−log10) of clinical feature significance is shown (based on chi-square test). *p < 0.05.

The univariate Cox analysis revealed 20 IICDLs associated with prognosis, with AL606489.1 and AC0794666.1 acting as protective factors and the remaining 18 acting as risk factors (**Figure 3A**). According to correlation analysis, most genes were related to one another (**Figure 3B**). Cluster analysis was performed on the 20 prognosis-related IICDLs. Patients with HCC clustered into two subgroups showed the best cluster effect, with good subgroup internal consistency and stability (**Figures 3C–F**). Heatmaps showed that the two clusters significantly differed in gene expression of IICDLs (**Figure 3G**). Cluster 2 had a better prognosis than Cluster 1 according to the survival analysis (**Figure 3H**). In Cluster 2, prognosis was significantly associated with male sex (p < 0.05), better T stage (p < 0.05), early clinical stage (p < 0.01), and early pathological grade (*p* < 0.05) (**Figures 4A–F**).

### Analysis and enrichment of differential expression of C1 and C2

**Figure 5:**
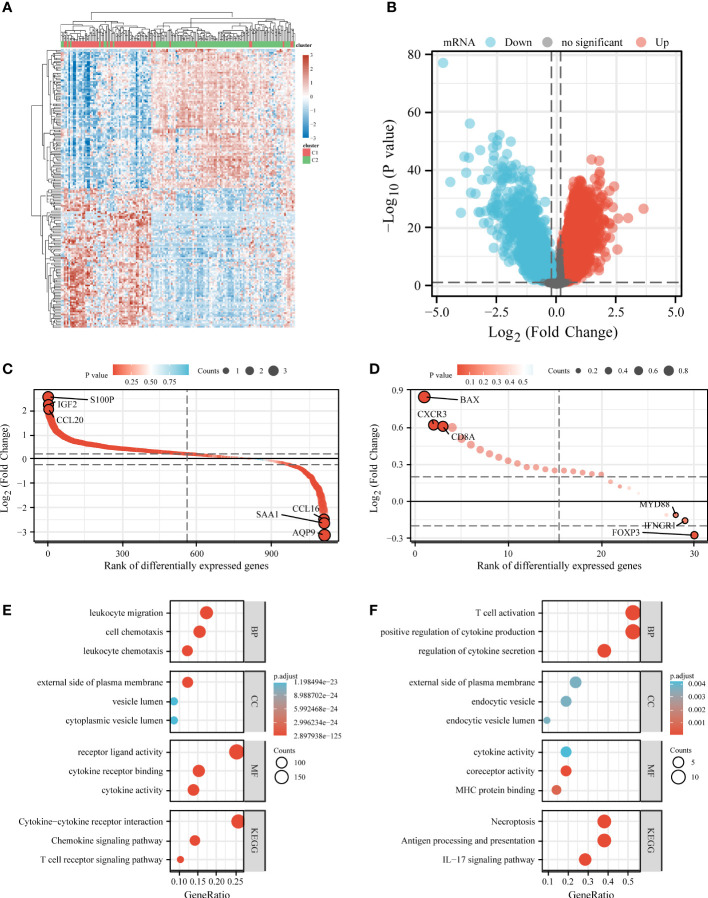
Differential expression and enrichment analysis of C1 and C2. Heat map **(A)** and volcano plot **(B)** showing differential gene expression between C1 and C2. Rank order plot showing differential expression of IRGs **(C)** and ICDRGs **(D)** in C1 and C2. KEGG and GO analysis of differentially expressed IRGs **(E)** and ICDRGs **(F)**. IRGs, immune-related genes; ICDRGs, immunogenic cell death-related genes.

We also analyzed differentially expressed genes in C1 and C2. Compared to C2, C1 had 1757 downregulated genes and 7901 upregulated genes (**Figures 5A, B**). Among 1794 IRGs, C1 had 569 upregulated genes (e.g., *S100P*), 713 downregulated genes (e.g., *AQP9*), and 512 unregulated genes (e.g., *SLP1*) in comparison to C2 (**Figure 5C**). Among 33 ICDRGs, C1 had 20 upregulated genes (e.g., *BAX*), one downregulated gene (*FOXP3*), and 12 unregulated genes (e.g., *PIK3CA*) in comparison to C2 (**Figure 5D**).

KEGG and GO analyses were performed based on differentially expressed IRGs and ICDRGs. KEGG analysis revealed that the differentially expressed ICDRGs were mainly involved in the IL-17 signaling pathway, necroptosis, and antigen processing and presentation (**Figure 5E**), whereas the differentially expressed IRGs were involved primarily in the cytokine–cytokine receptor interaction, chemokine signaling pathway, and T cell receptor signaling pathway (**Figure 5F**). GO analysis suggested that the differentially expressed ICDRGs were mainly involved in T cell activation, endocytic vesicle, and MHC protein binding (**Figure 5E**), whereas the differentially expressed IRGs were primarily involved in leukocyte migration, the external side of the plasma membrane, and cytokine receptor binding (**Figure 5F**).

### Correlation analysis between clusters and immune status, ICB response, and chemotherapy response

**Figure 6:**
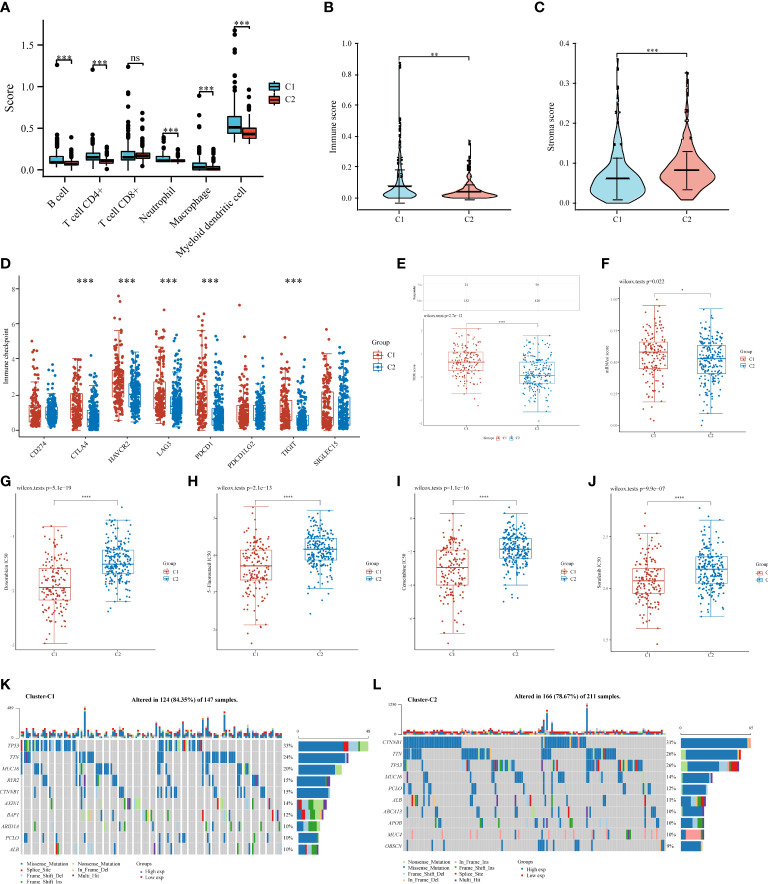
Comparisons of immune status, stemness, ICB response, and chemotherapy response between C1 and C2. Box plots present differential immune infiltration **(A)**, immune score **(B)**, stroma score **(C)**, immune checkpoint gene expression **(D)**, TIDE score **(E)**, mRNAsi score **(F)**, doxorubicin IC50 **(G)**, 5-fluorouracil IC50 **(H)**, gemcitabine IC50 **(I)**, and sorafenib IC50 **(J)**. Oncoprint visualization of the top 10 most commonly mutated genes in C1 **(K)** and C2 **(L)**. mRNAsi, mRNA expression-based stemness index. (*P < 0.05; **P < 0.01; ***P < 0.001; ****P < 0.0001; ns, not significant).

We used the TIMER algorithm, the only method that considers tissue specificity when estimating immune cell populations (61), to determine whether there was a difference in immune infiltration between the two clusters. We found significant differences in the CD4^+^ T cells (p < 0.001), neutrophils (p < 0.001), macrophages (p < 0.001), B cells (p < 0.001), and myeloid dendritic cells (p < 0.001), suggesting that C2 exhibited stronger immunosuppression than C1 (**Figure 6A**). The presence of stromal and immune cells in tumor tissues is indicated by stromal and immune scores; the interaction of cancer cells and tumor stroma affects cancer development, facilitates metastasis, and evades immune surveillance. Therefore, we further investigated the immune and stroma score differences between C1 and C2, revealing that C1 had a higher immune score and a lower stroma score than C2 (**Figures 6B, C**). Additionally, we used the ggplot2 package (v3.3.3) to analyze the ICGs in C2 and C1 and found that *CTLA4*, *HAVCR2*, *LAG3*, *PDCD1*, and *TIGIT* were downregulated (**Figure 6D**, p < 0.001). TIDE scores for C1 were significantly higher than those for C2 (p < 0.0001), indicating that C2 might achieve greater clinical benefit with ICBs (**Figure 6E**).

Stemness, a molecular marker associated with stem cells, has emerged as a valuable predictor or prognostic factor (51–53). A higher stemness in C1 was noted (**Figure 6F**), suggesting a poorer prognosis for patients in C1. Notably, tumor stemness considerably contributes to cancer chemotherapy resistance (62). Therefore, we assessed whether C1 and C2 responded differently in recommending chemotherapeutic drugs for the treatment of HCC, such as doxorubicin (55), 5-fluorouracil (56), gemcitabine (57), and sorafenib (58). C1 was associated with lower IC_50_ levels for doxorubicin (*p* < 0.0001), 5-fluorouracil (*p* < 0.0001), gemcitabine (*p* < 0.0001), and sorafenib (*p* < 0.0001) (**Figures 6G**–**J**), suggesting that chemotherapy may have more beneficial on C1 than on C2. Additionally, we analyzed somatic mutations in C1 and C2 and found that C1 had a high frequency of *TP53* (33%), *TTN* (24%), and *MUC6* (20%) (**Figure 6K**), whereas C2 had a high frequency of *CTNNB1* (31%), *TTN* (26%), and *TP53* (26%) (**Figure 6L**).

### Developing an IICDL-related prognosis signature in TCGA-HCC cohort

**Figure 7:**
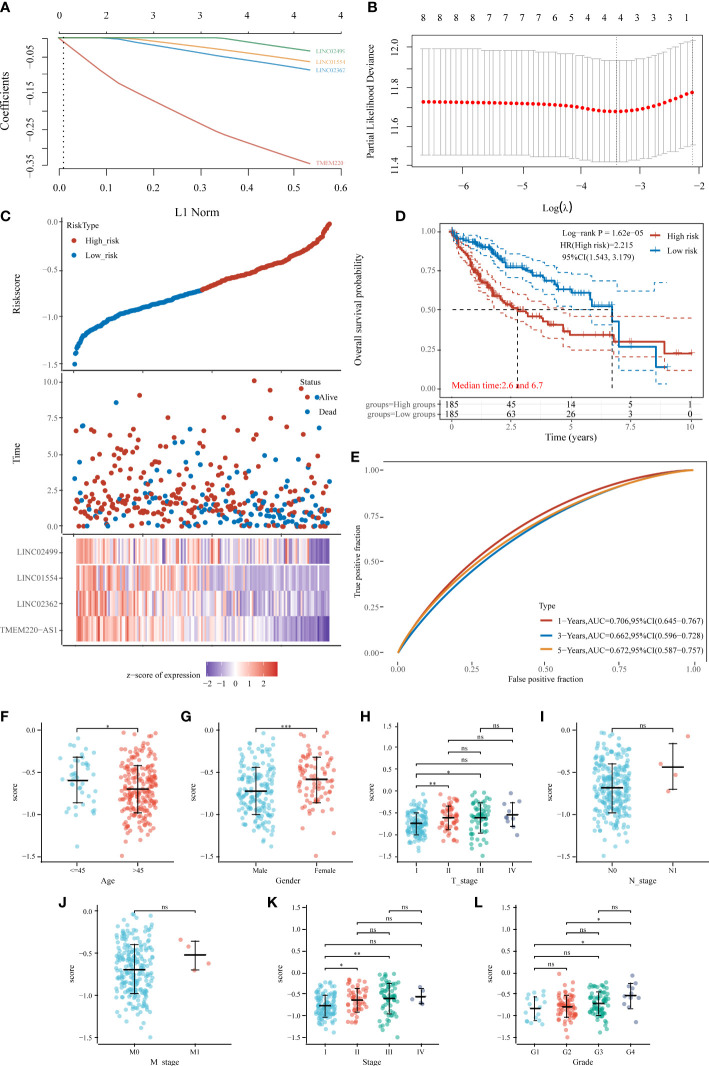
Prognostic model of HCC based on 20 IICDLs. **(A)** LASSO coefficient profiles of the four IICDLs. **(B)** A plot of the error rates from ten-fold cross-validation. **(C)** An overview of the risk score distribution, survival status of each patient, and a heat map of four IICDLs. **(D)** KM survival curve illustrating the predictive value of the risk model. **(E)** ROC curve of the predictive value of the risk model. Correlations between risk score and age **(F)**, sex **(G)**, T stage **(H)**, N stage **(I)**, M stage **(J)**, clinical stage **(K)**, and grade **(L)**. (*P < 0.05; **P < 0.01; ***P < 0.001; ns, not significant).

We then built a prognostic model based on 20 prognosis-related IICDLs. Four IICDLs were tested and chosen for the prediction model based on the results of the LASSO regression analysis (**Figures 7A, B**). Risk score models were calculated based on the following algorithm: risk score = (−0.2854) * TMEM220-AS1 + (−0.0614) * LINC02362 + (−0.0418) * LINC01554 + (−0.0114) * LINC02499. Patients with HCC were divided into two groups based on their risk scores. **Figure 7C** shows the distribution of risk scores, survival status, and gene expression for these four genes. The KM curves showed that the OS for high-risk patients was significantly worse than that for low-risk patients (p < 0.0001, HR = 2.215) (**Figure 7D**).

The area under curves (AUCs) of time-dependent ROC curves for 1-, 3-, and 5-year OS were 0.706, 0.662, and 0.672, respectively (**Figure 7E**), indicating a good predictive performance. The AUCs of the IICDL-based prognostic model were 0.75, 0.77, and 0.77 for the 1-, 3-and 5-year survival times, respectively.

Furthermore, we identified the differences in risk scores between subgroups based on different clinical pathological factors. The risk score was significantly associated with age (*p* < 0.05), sex (*p* < 0.001), T stage (*p* < 0.05), clinical stage (*p* < 0.05), and pathological grade (*p* < 0.05) (**Figures 7F**–**L**).

### Developing a predictive nomogram for OS prediction

**Figure 8:**
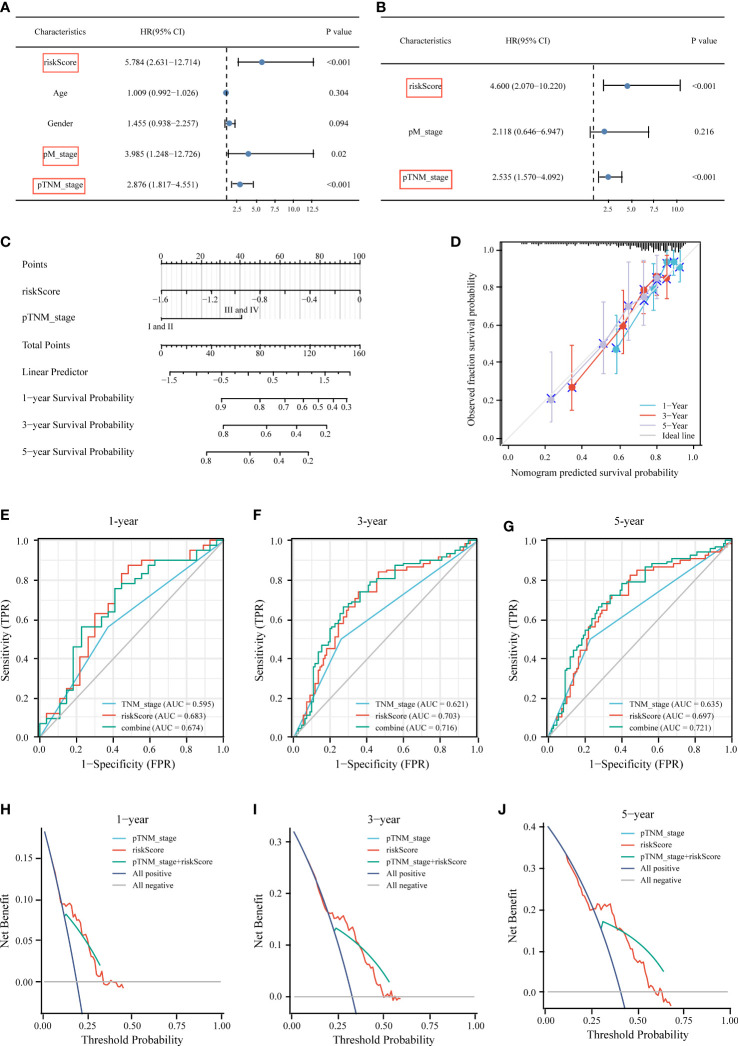
Construction of the nomogram for predicting OS of HCC patients in TCGA cohort. Univariate **(A)** and multivariate **(B)** forest plots of the risk score model and clinicopathological characteristics associated with overall survival. **(C)** The nomogram was constructed based on two independent prognostic factors. **(D)** Calibration plot for internal validation of the nomogram. Time‐dependent ROC **(E–G)** and DCA curves **(H–J)** of the TNM stage model, risk score model, and the combined model for 1‐, 3‐ and 5‐year OS prediction.

We performed univariate and multivariate Cox regression analyses to determine whether other traditional clinical characteristics affected the prognostic model. The TNM stage (*p* < 0.001, HR = 2.535) and risk score (*p* < 0.001, HR = 4.6) were independent prognostic factors for OS (**Figures 8A, B**). A predictive nomogram was built to assist in the accurate prediction of clinical outcomes (**Figure 8C**). The predicted OS outcomes matched the actual observations more closely, according to the calibration plot for the internal validation of the nomogram (**Figure 8D**). Additionally, the predictive accuracy of the nomogram and individual prognostic factors were compared using time-dependent ROC curves. The AUCs of the nomogram at 1-, 3-, and 5-year OS were 0.674, 0.716, and 0.721, respectively, which were better than those of the models with only one independent factor (**Figures 8E–G**). DCA was used to determine the clinical relevance of these models, with the combined model predicting outcomes with the highest accuracy (**Figures 8H–J**).

### Correlation of immune status, MSI score, ICB response, stemness, and chemotherapy response with IICDL signature

**Figure 9:**
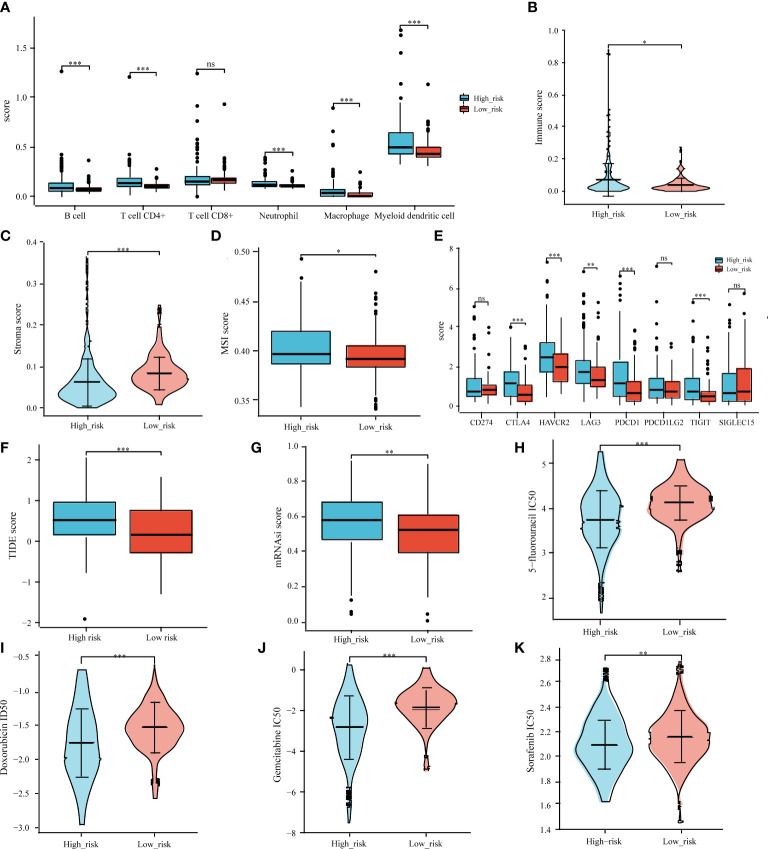
Correlation analysis of prognostic signature and tumor immune cell infiltration, immune checkpoint inhibitors, ICB response, MSI, tumor stemness, and common chemotherapeutic agents. Associations between the IICDL signature and tumor immune cell infiltration **(A)**, immune score **(B)**, stroma score **(C)**, MSI score **(D)**, immune checkpoint inhibitors **(E)**, TIDE score **(F)**, mRNAsi score **(G)**, and IC_50_ of chemotherapeutic drugs **(H–K)**. (*P < 0.05; **P < 0.01; ***P < 0.001; ns, not significant).

Previous research has linked different clusters to immune status, ICB response, stemness, and chemotherapy response. Accordingly, we aimed to determine whether there were any significant differences between the high-risk and low-risk groups. The TIMER algorithm was applied to investigate the relationship between the tumor immune microenvironment and the signature. The high-risk group had more extensive infiltration of immune cells, including B cells (*p* < 0.0001), CD4^+^ T cells (*p* < 0.0001), neutrophils (*p* < 0.0001), macrophages (*p* < 0.0001), and myeloid dendritic cells (*p* < 0.0001), than that in the low-risk group (**Figure 9A**). We also compared the immune, stroma, and MSI scores between the risk groups and found that high-risk patients had lower immune and stroma scores (**Figures 9B–D**) and expressed high levels of five immune checkpoint inhibitors (CTLA4, HAVCR2, LAG3, PDCD1, and TIGIT) (**Figure 9E**). A higher TIDE score was obtained in the high-risk group (**Figure 9F**). The OCLR algorithm indicated a higher stemness in the high-risk group (**Figure 9G**).

We also investigated whether the high-risk and low-risk groups responded differently to doxorubicin, 5-fluorouracil, gemcitabine, and sorafenib. The high-risk group had lower IC_50_ levels for doxorubicin (*p* < 0.001), 5-fluorouracil (*p* < 0.001), gemcitabine (*p* < 0.001), and sorafenib (*p* < 0.01) (**Figures 9H–K**), indicating that chemotherapy may have a greater impact on high-risk patients.

### Biological validation and independent prognostic analysis of IICDL expression

**Figure 10:**
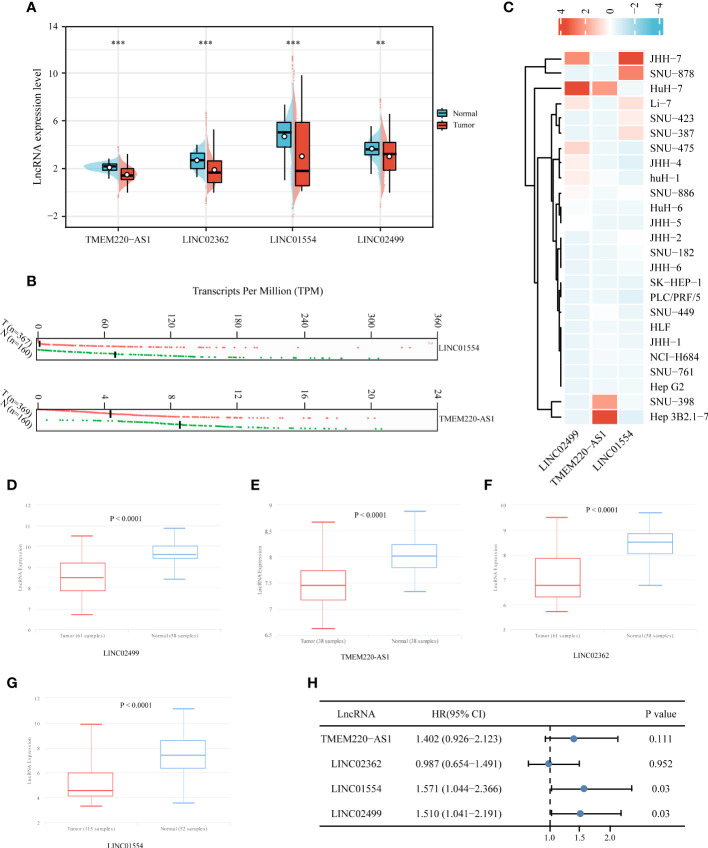
Expression validation and independent prognostic analysis of four IICDLs. IICDL expression in HCC tumor tissues and healthy tissues according to TCGA **(A)** and GEPIA **(B)** databases. **(C)** IICDL expression in HCC cell lines according to the CCLE database. **(D–G)** IICDL expression in HCC tumor tissues and normal tissues according to the lnCAR database. **(H)** Multivariate Cox regression analysis of the four IICDLs for prognosis prediction of patients with HCC. (**P < 0.01; ***P < 0.001; ns, not significant).

To verify IICDL expression, we collected data from HCC patients and cell lines. TCGA data revealed a significant decrease in the expression levels of TMEM220-AS1, LINC02362, LINC01554, and LINC02499 in HCC samples. (**Figure 10A**). According to the GEPIA database analysis, LINC01554 and TMEM220-AS1 were downregulated in HCC (**Figure 10B**). CCLE data indicated that LINC02499, TMEM220-AS1, and LINC01554 were expressed at low levels in most HCC cell lines (**Figure 10C**). The lnCAR database further confirmed these results. TMEM220-AS1, LINC02362, LINC01554, and LINC02499 were significantly downregulated in HCC tissues compared to that in the healthy liver tissues (*p* < 0.0001) (**Figures 10D–G**). Finally, the effect of single genes on HCC prognosis was analyzed using multivariate Cox regression analysis. The results suggest that LINC01554 and LINC02499 could be used as independent risk factors for determining HCC patient prognosis (**Figure 10H**).

## Discussion

This study revealed an IICDL-related signature comprising four lncRNAs that could predict clinical outcomes and treatment responses in patients with HCC. Our proposed model predicted the survival of patients with HCC accurately.

Various types of immunotherapies and new chemotherapy modalities rely on tumor immunogenicity for their success (63). Surprisingly, certain types of stress can cause specific cells to initiate a proinflammatory process, increasing T cell activation, a process known as ICD. ICD has been identified to trigger adaptive immune responses by releasing danger-associated molecular patterns, which play an immunogenic role when they reach the tumor microenvironment (TME) (64). Hence, we speculated that patients with HCC may benefit from ICD occurring in the TME and that an ICD/immune lncRNA model would be an accurate predictor of patient prognosis and medication response.

As a first step, DEirlncRNAs and DEidcrlncRNAs were identified from TCGA data. Intersections of the two lncRNA sets were referred to as IICDLs. Of the 81 IICDLs, 20 were significantly associated with prognosis of patients with HCC, namely, AL606489.1, AC004160.1, AC099508.2, TMEM220-AS1, AC079061.1, AC115619.1, LINC02754, LINC02037, AC008549.1, AP001065.3, LINC01018, LINC00261, LINC02362, AC009093.5, AC079466.1, LINC01554, AC010280.2, AP001781.1, LINC02499, and AL161645.2. Among them, AC079466.1 (65), LINC01554 (66), LINC00261 (67–69), LINC02499 (70–73), and AC008549.1 (74) were closely correlated with the prognosis of patients with HCC. In addition, patients with HCC had low levels of TMEM220-AS1, a protein that inhibits malignant behavior by increasing TMEM220 expression and inactivating Wnt/β-catenin (75). Another study found that TMEM220-AS1 promoted metastasis and proliferation in HCC through the microRNA(miR)-484/MAGI1 axis (76). Further, TMEM220-AS1 was found to be upregulated in gliomas and positively correlated with their progression (77). LINC01554 has been identified as a significant lncRNA involved in the pathogenesis of esophageal cancer and nonalcoholic fatty liver disease (78, 79). Additionally, LINC01554 inhibits the glycolytic action of cells in hepatocellular carcinoma, suppressing tumor growth (67). LINC01018 was poorly expressed in HCC; however, sponging miR-182-5p conferred a novel tumor suppressor role (80). LINC02362 suppressed HCC growth by modulating miR-516b-5p and SOSC2 (81). LINC02499 inhibits the proliferation, migration, and invasion of hepatocellular carcinoma cells (70). These results are consistent with those of ours.

Precision medicine is emerging as a powerful clinical strategy in oncology, with the goal of improving clinical outcomes and patient progression (82). Genotyping patients and targeting therapies are key components of precision medicine. Consequently, we divided patients with HCC into two clusters, C1 and C2, according to the 20 IICDLs. There were significant differences between HCC patients in C1 and C2 in terms of prognosis, clinical stage, histopathological grade, and ICG expression. Specifically, patients in C2 had a higher immune score, higher ICB response, and lower stemness score than those in C1. Furthermore, patients in C1 and C2 responded differently to doxorubicin, 5-fluorouracil, gemcitabine, and sorafenib. Notably, these findings are consistent with those of a previous study, reporting that HCC patients with high immune infiltration and ICB response had a longer OS (83).

Four IICDLs (TMEM220-AS1, LINC02362, LINC01554, LINC02499) were identified and used to develop prognostic models for HCC patients using LASSO Cox analysis. Patients with HCC were divided into high- and low-risk groups based on risk scores for OS. We found that the high-risk group of HCC patients had a worse prognosis than the low-risk group. The four-lncRNA signature had an independent effect on HCC prognosis, and the model had a better predictive performance. Previous studies have reported similar results. Hong et al. developed an immune-related lncRNA signature to predict the prognosis of HCC patients (84). Another prognostic signature based on immune- and ferroptosis-related lncRNAs could predict the prognosis and immune infiltration of HCC (78). In addition, cuproptosis-related lncRNA signatures predicted the patient outcomes and response to ICB therapy (79). Our study also examined the correlation between prognostic risk scores and HCC patient characteristics. The risk score increased along with the T stage, clinical stage, and pathological grade. Overall, the prognostic values of the IICDL signature in our study for patients with HCC were consistent with the results of previous studies.

We also compared the immune score, ICGs, TIDE score, mRNAsi score, and chemotherapy response between the high- and low-risk groups. The high-risk group had low immune cell infiltration, low ICG expression, poor response to ICB therapy, and high stemness. The high-risk and low-risk groups showed significantly different reactions to several chemotherapy drugs (doxorubicin, 5-fluorouracil, gemcitabine, and sorafenib). Interestingly, low macrophage (85) and NK cell (86) infiltration predicted a poor prognosis in patients with HCC. High levels of ICGs, such as CTLA4 (87) and LAG3 (88), had a negative impact on the prognosis of HCC patients. HCC patients with high stemness genes tend to have more aggressive tumor growth with poor prognoses (89). Taken together, these findings were consistent with ours.

According to the molecular subtypes and prognostic signatures constructed from prognostic IICDLs, personalized treatment should depend on molecular subtypes and risk groups. Using the TIDE algorithm, for instance, patients in the low-risk group and with the C2 molecular subtype might benefit more from immunotherapy. High-risk group and C1 were suggestive of a large number of immune cells (B cells, CD4^+^ T cells, neutrophils, macrophages, and myeloid dendritic cells) and a higher expression of ICGs (PD-L1, CTLA-4, TIGIT, HAVCR2, LAG3, PDCD1LG2, and SIGLEC15), indicating that the high-risk group and C1 exhibit resistance to immunotherapy. This probably explains the poor prognosis of patients with HCC in the high-risk group and with the C2 molecular subtype. In fact, overexpression of the PD-L1 surface molecule inhibited T cell responses by engaging PD-1 on the surrounding T lymphocytes, thereby promoting the progression and diffusion of cancer (90, 91). PD-L1 is also expressed by macrophages and interacts with PD-1 on cytotoxic T cells, contributing to the escape of tumor cells from the immune system (92, 93). The overexpression of CTLA-4/TIGIT on T lymphocytes and NK cells also plays an important role in immune escape (94, 95). A recent study found that SIGLEC15 glycosylation promoted tumor growth and immune escape (96). These findings suggest that the molecular subtypes and prognostic signatures we identified may be useful for the implementation of future immunotherapy strategies.

This study had some limitations. First, our signature was not verified in another database. LncRNAs obtained from TCGA may differ from those in other databases owing to differences in chip technology and recording method. Our search of the ICGC database did not yield any corresponding expression of the IICDLs. Second, although we validated IICDL expression in the GEPIA, CCLE, and lnCAR databases, *in vitro* experiments may provide more convincing results. Since fresh tissue samples are required for detecting lncRNA expression, it was impossible to collect sufficient survival time within a short period. Our follow-up research will focus on the analysis of bioinformatic and clinical data.

## Conclusions

Overall, we identified molecular subtypes based on IICDLs in HCC and constructed a prognostic signature using IICDLs. Different molecular subtypes and risk groups were also analyzed for clinical characteristics, immune landscapes, ICB responses, stemness, and chemotherapy responses. In the future, the proposed signature may provide clinical evidence to support decisions regarding HCC patient treatment and prognosis.

## Data availability statement

The raw data supporting the conclusions of this article will be made available by the authors, without undue reservation.

## Author contributions

ZL, WG, and JL conceived and designed the study. MH and YG collected the data, performed bioinformatics analysis, and wrote the initial draft of the manuscript. MH, WG, and YL analyzed the data and generated the figures. ZL conceptualized and revised the manuscript. All authors contributed to the article and approved the submitted version.

## Funding

This work was supported by the Natural Science Fund of Shandong Province (No. ZR202010220061), and Medical Discipline Construction Project of Pudong Health Committee of Shanghai (No. PWYst2021-18).

## Acknowledgments

All authors are to be commended for their collaboration.

## Conflict of interest

The authors declare that the research was conducted in the absence of any commercial or financial relationships that could be construed as a potential conflict of interest.

## Publisher’s note

All claims expressed in this article are solely those of the authors and do not necessarily represent those of their affiliated organizations, or those of the publisher, the editors and the reviewers. Any product that may be evaluated in this article, or claim that may be made by its manufacturer, is not guaranteed or endorsed by the publisher.
